# Transactivation Response DNA-Binding Protein of 43 (TDP-43) and Glial Cell Roles in Neurological Disorders

**DOI:** 10.7759/cureus.30639

**Published:** 2022-10-24

**Authors:** Hussain Hussain, Tamara Djurin, Jacqueline Rodriguez, Lia Daneelian, Sardar Sundi, Aya Fadel, Zahraa Saadoon

**Affiliations:** 1 Internal Medicine, Larkin Community Hospital, Miami, USA; 2 Internal Medicine, Ross University School of Medicine, Miramar, USA; 3 Internal Medicine, Medical University of the Americas, Devens, USA; 4 Internal Medicine, University of Medicine and Health Sciences, New York, USA; 5 Internal Medicine, Hackensack Meridian Ocean Medical Center, Brick Township, USA; 6 Internal Medicine, University of Baghdad School of Medicine, Baghdad, IRQ

**Keywords:** myelination, amyotrophic lateral sclerosis, glycogen, neurological disorders, dementia, microglia, alzheimer's disease, astrocytes, gliosis, tdp-43

## Abstract

The trans-activation response DNA-binding protein of 43kDa (TDP-43) is involved in the pathogenesis of multiple brain disorders. As scientists are unraveling TDP-43 function and its impact on various diseases, we have begun to subcategorize them into TDP-43 proteinopathies. Furthermore, glial cell dysfunction contributes to various disorders, and TDP-43 is involved with glial cells via multiple pathways (direct or indirect) that aggravate the pathophysiology of such disorders. We are only now discovering and understanding the vast and diverse roles TDP-43 plays on neuronal cells and its effects on gliosis and neurodegenerative pathologies. It has multiple roles: mRNA maturation and splicing, transporting and maintaining mRNA stability, a component of stress granules and ubiquitination of dysfunctional or misfolded proteins, transcription of microtubule “Futsch” protein, and a role in maintaining synapse integrity and possibly more as we continue to research and uncover the labyrinth of the neuronal network. TDP-43 could also have a detrimental impact on glial cell activation and pathophysiology in diseases where TDP-43 is associated with its pathogenesis. We will review the pathophysiology of various neurological disorders that are associated with the alteration of the TDP-43 levels along with glial cell activation. Further, multiple diseases have glial cell participation in the pathogenesis, and the role of TDP-43 has not yet been investigated. We, therefore, explore those disorders in the context of both TDP-43 and glial cells involvement. This step will enhance the understanding of neurodegeneration where further research could prompt curative modalities with the advancement of technology.

## Introduction and background

There are two important brain-related structures that have contributed to different pathological processes discovered in the last decade. Initially, the trans-activation response DNA-binding protein of 43kDa (TDP-43) had been discovered in 1995 as a suppressor protein for HIV genes, it’s critical for the regulation of the viral gene expression [1]. The structure of TDP-43 consists of 414 amino acids and is encoded by the TAR DNA Binding Protein (TARDBP) gene on chromosome 1 [2]. Various mutations identified in the last decades in TARDBP led to pathogenesis of multiple disorders [2]. According to the structure, the N terminal has a nuclear signal and two ribonucleic acid (RNA) recognition motifs, while the C terminal has a glutamine/asparagine-rich domain, as well as a glycine-rich domain [2].

The physiological functions of TDP-43 are complicated. Extensive research has been conducted in the last few decades to understand the main physiological processes that aid in identifying future pathological disorders. TDP-43 contributes to RNA transcription, translation, messenger ribonucleic acid (mRNA) transport, splicing, and mRNA stabilization, as well as the processing of long non-coding RNA and microRNA [3]. The TDP-43 can be predominantly localized within the nucleus, cytoplasm, or even within the mitochondria [3,4]. In fact, imbalance of TDP-43 leads to malfunction of RNA and disturbance of neuronal integrity as well as the function. The location of TDP-43 within the nucleus provides specific regulatory functions for both transcription and splicing (e.g. CFTR, Huntingtin, amyloid precursor protein (ALS), alpha-synuclein, etc) [3,4]. Therefore, an imbalance of TDP-43 results in dysfunction of neurons because of dysregulation of the splicing and transcription [3-5]. Furthermore, TDP-43 plays an essential role in mRNA maturation via interaction with specific mRNA sequences [5]. Also, the TDP-43-RNA complex produces specific granules called ribonucleoprotein to transport mRNA with the aid of microtubules such as in ALS, which occurred due to the failure of transportation of ribonucleoprotein granules due to mutant TDP-43 [5,6]. In addition, TDP-43 regulates mRNA translation by forming a particular complex with other proteins including ribosomal protein, and receptor for activated C kinase 1 [5,6]. Moreover, stress granule formation is another function of TDP-43 that occurs during a stressful environment to protect the neurons. This reversible function provides safe storage for RNA binding proteins [5,6]. Also, TDP-43 plays a critical role in protecting the neurons via assembling and maintaining those granules. Another function of TDP-43 is the biogenesis of non-coding RNAs (microRNA), which in turn regulate gene expressions via nuclear enriched abundant transcript 1 (NEAT-1) [5-7].

Glial cells are non-neuronal cells that do not participate in impulse transmission. These cells have important functions to maintain the environment safe and suitable for the neurons by providing protection, supportive mechanisms, regulation of microcirculation, synaptic plasticity and pruning, memory, and maintaining synaptic transmission through electrolytes and neurotransmitter regulations [8]. They are subclassified into two categories; macroglia (astrocytes, ependymal cells, oligodendrocytes, and NG2-glia), and microglia which are phagocytic [8,9]. In terms of synaptic formation and function; microglia and astrocytes are playing essential roles in these functions. Premature synapses develop when either/both of these cells are dysfunctional. Astrocytes secrete specific components such as neuroligin, ephrin-A-3, D-serine, ephrin-B-1 (abnormal level linked to synaptic dysfunction and decrease glutamatergic signals), and thrombospondin that are important in excitatory synapses formation [8-10]. Also, astrocytes can regulate lactate, adenosine triphosphate (ATP), adenosine, and glutamate levels. Synaptic pruning is another important function that is under the control of microglia. This aids in neuronal transmission which results in improving memory and learning function [8-11]. Ephrin-B-1 is activated in astrocytes and sends signals to activate ephrin-B-1 receptors on microglia [8-11]. Therefore, there is a correlation between astrocytes and microglia that could lead to further research to discover different pathways among them.

Oligodendrocytes are essential cells for myelination, and astrocytes play an important role in this process as well [12]. Astrocytes facilitate oligodendrocyte progenitor cell proliferation, differentiation, and protection, as well as enhance the contact between the oligodendrocyte and the axon [12]. These functions are accomplished via several signals including fibroblast growth factor, insulin-like growth factor, platelet derived growth factor, leukemia inhibitory factor-like protein, neuregulin-1, gamma-secretase, osteopontin, ciliary neurotrophic, and neurotrophin-3 [13]. Also, a recent study showed any physical contact between astrocytes and oligodendrocytes increases the maturation of oligodendrocytes [14]. There are different mechanisms of communication among those cells that are not well understood yet.

Ependymal cells are lining the ventricular system of the brain, as well as the spinal cord canal that transmits the cerebrospinal fluid (CSF) [15]. Their functions are very critical to the central nervous system (CNS); initially, ependymal cells produce the CSF through microvilli at the apical surface, and it also facilitates the movement of the CSF by their cilia [15]. Secondly, ependymal cells migrate and activate the forebrain in case of a stroke to aid in regenerative function [15]. Also, an animal study by Nelson et al. (2019) confirmed the relationship between spinal cord injury and the regenerative function of ependymal cells with the aid of glucocorticoid receptors located on the ependymal surface [15].

## Review

Mechanism of TDP-43 neurodegeneration

**Figure 1:**
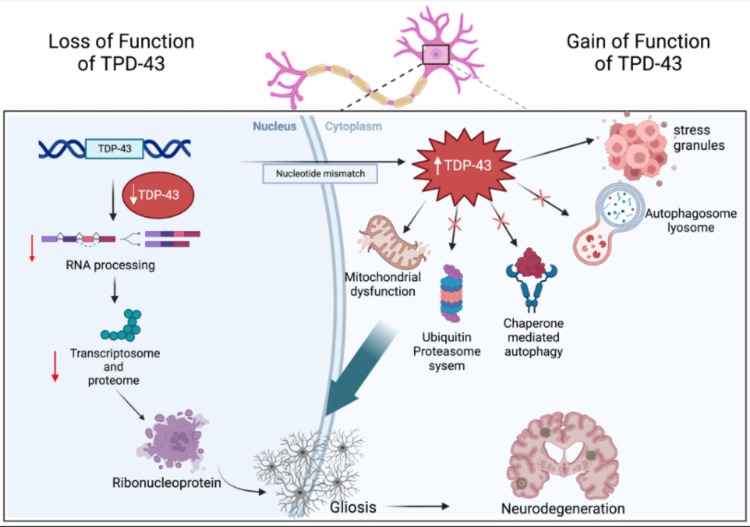
The roles of trans-activation response DNA-binding protein of 43kDa (TDP-43) TDP-43 is an RNA binding protein containing two RNA-recognition motifs, a nuclear localization signal, and a nuclear export signal. The protein is normally concentrated in the nucleus and also shuttles back and forth between the nucleus and cytoplasm. When there is a loss of function of TDP-43 levels decrease in the nucleus and thus a subsequent decrease in transcriptomes, proteasomes, and ribonucleoproteins. Leading to gliosis and neurodegeneration. During the event of unrepaired nucleotide mismatch, there are increased levels of TDP-43 in the cytoplasm. Normal degradation pathways such as ubiquitin-proteasome system, chaperone-mediated autophagy, and autophagosome-lysosome degradation are unable to maintain function with the increased TDP-43 in the cytoplasm. The mitochondria additionally become dysfunctional and stress granules form leading to gliosis and ensuing neurodegeneration. The authors have designed the figure based on the scientific information provided in references [16-21].

TDP-43 has been found as a major proponent of multiple pathways (Figure 1) including providing functionality in the ubiquitin-proteasome system, chaperone-mediated autophagy in lysosomes, autophagosome-lysosome activation, stress granule formation, and inducing mitochondrial dysfunction.

In the initial pathway the of ubiquitin-proteasome system (UPS), misfolded proteins and/or dysfunctional proteins are ubiquitinated as a tag and sent to the proteasome to be degraded [16]. If this becomes altered it increases the risk and promotes aggregation and accumulation of misfolded/dysfunctional proteins with subsequent disease formation. In our discussion of neurodegenerative diseases, this will be seen in the clinical presentation of patients with their respective diseases.

The next major pathway in which the health of TDP-43 is imperative for the proper function is chaperone-mediated autophagy in lysosomes (Figure 1) [16]. The function of this pathway is for the degradation of cytosolic proteins in the lysosome [16]. A chaperone-mediated autophagy substrate tags the proteins and then gets sent to the lysosome for degradation [16,17]. In the similar format of the UPS, if this mechanism is no longer functional in any capacity the proteins are not degraded, instead, aggregate and accumulate, therefore, neurodegeneration occurs [16,17].

In the third mechanism that requires TDP-43 health, the autosome-proteasome pathway proteins are degraded keeping normal function [16,17]. The autophagosome-lysosome pathway degrades dysfunctional or unused intracellular macromolecules [16,17]. The mechanism is performed through the inhibition of mammalian target of the rapamycin complex 1 (mTORC1) and activation of transcription factor EB (TFEB). A phagosome is produced, consumes the macromolecules, and breaks them down [16,17]. The products can then be used for cellular energy building and or building blocks for other macromolecules [16,17]. This pathway has also been shown to degrade aggregated proteins. Therefore, the lack of proper function of the autophagosome-lysosome pathway will allow for aggregated misfolded proteins, which are found in major neurodegenerative diseases [16,17]. More specifically, the aggregation of TDP-43 causes loss of function and is linked to the mTORC1 activation and TFEB activation [16,17]. It leads to impairment of the mTORC1 resulting in the accumulation of autophagic vacuoles contributing to TDP-43 aggregation and neurodegeneration (Figure 1) [16,17].

Fourthly, the stress granule formation occurs following an external stimulus in the cytoplasm. Stress granule formation is important to cell survival following stressors in the environment [18,19]. The granules are membrane-less organelles believed to be essential for cell survival through the storage of non-essential mRNAs, translation factors, and RNA-binding proteins when cells undergo stress [18,19]. Initially, they assemble structures at the beginning of stress and eventual disassembly as the cell recovers [18,19]. Stress granule formation has been observed in neurodegenerative diseases such as amyotrophic lateral sclerosis (ALS) and frontotemporal lobar degeneration (FTLD). In the transformed cell line depletion of TDP-43 negatively impacted the steps in which stress granules form [18,19]. In an experiment conducted, a reduction in TDP-43 levels results in an acceleration of stress granule disassembly in astrocytes and cortical neurons [18,19]. Thus, elucidating a key role in which TDP-43 regulation contributes to the proper function of stress granule formation (Figure 1) [19].

Lastly, there is growing evidence to understand TDP-43 roles in mitochondrial homeostasis and the defect causing cellular stress [20]. Mitochondrial damage has been observed across multiple TDP-43 proteinopathies (Figure 1) [20]. It has been associated with membrane damage and elevated reactive oxygen species (ROS) as TDP-43 localization to the mitochondria suppresses complex I activity and ultimately decreases ATP production [20]. Additionally, mitochondrial unfolded protein response (UPRmt) is responsible for detecting dysfunctional proteins and maintaining mitochondrial integrity with the help of Lon protease (LonP1), a UPRmt protease [20]. Increased TDP-43 mitochondrial aggregation has shown to increase activation of UPRmt and upregulation of LonP1 [20]. These mechanisms are in place to repair mitochondrial stress in the early changes, but chronic production of ROS from TDP-43 leads to irreversible mitochondrial damage and dysfunction [20]. This activates the lysosome and autophagosome mechanism to expel the dysfunctional mitochondria leading to the neuroinflammatory cascade [20,21].

All the above pathways can be linked to TDP-43 dysfunction and the subsequent pathway being rendered dysfunctional causing subsequent protein aggregation and accumulation (Figure 1). The protein aggregation and accumulation occur in the neuronal cells found in the brain, such as glial cells, astrocytes, and oligodendrocytes [19-21]. The protein aggregates appear to be toxic to neuronal cells causing injury and subsequent death [19-21]. The correlation that is commonly found between aggregation and neurodegeneration is that as protein aggregation increases neurodegeneration increases as well [19-21]. The cells in which this occurs are in the brain, i.e glial cells [19-21]. When glial cells have protein aggregation due to misfolding or accumulation, it gets signaled to other neighboring glial cells [19-21]. It is proposed this occurs through cell-to-cell signaling and/or through glial cells triggering a pro-inflammatory cascade that causes glial cell death [19-21]. We will discuss various neurodegenerative diseases in the following paragraphs.

Gliosis in TDP-43 proteinopathies

**Figure 2:**
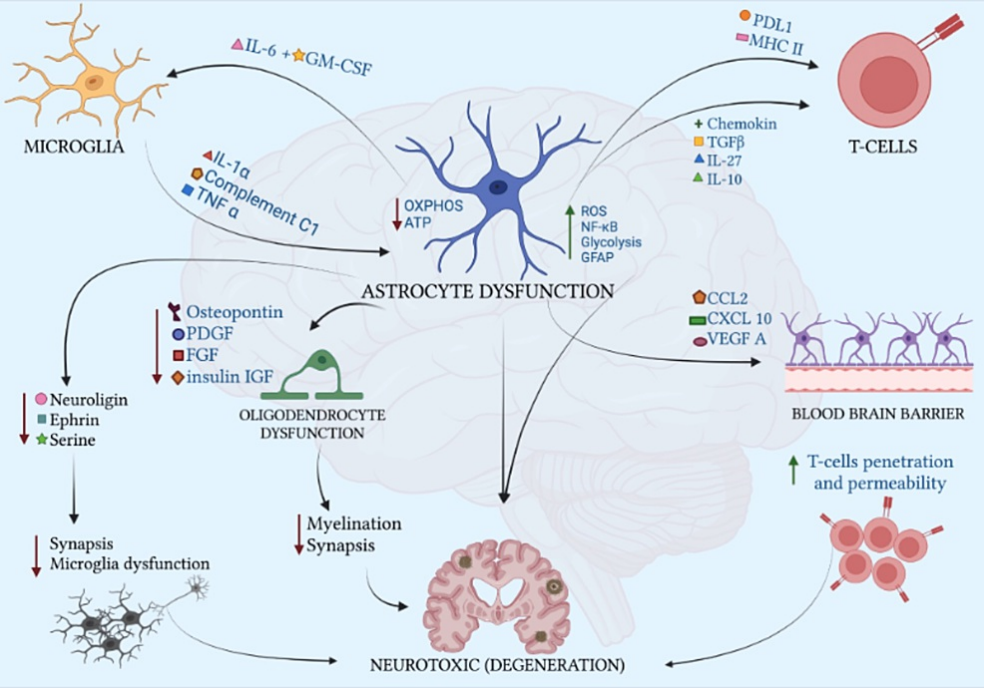
Gliosis Astrocytes facilitate cell proliferation, differentiation, and protection, and enhance the contact between the oligodendrocytes and the axon. The normal function of astrocytes is neuroprotective, however, when rendered dysfunctional they become neurotoxic. Increasing reactive oxygen species (ROS), nuclear factor- kappa B (NF-κB), glycolysis, and glial fibrillary acidic protein (GFAP). While oxidative phosphorylation and ATP decrease. Astrocytes release pro-inflammatory cytokines such as programmed cell death ligand 1 (PD L1), MHC II, Chemokin, transforming growth factor- beta (TGF-β), interleukins 27 (IL-27), and interleukin- 10 (IL-10) and act on T-cells via TCR. Astrocyte dysfunction affects the blood-brain barrier increasing permeability and T-cell penetration. The cytokines affecting the blood-brain barrier include chemokine ligand 2 (CCL2), C-X-C chemokine ligand 10 (CXCL 10), and vascular endothelial growth factor A (VEGF A).  Oligodendrocyte dysfunction additionally occurs leading to a decrease in myelination and synapsis as the neurons are unable to communicate with one another, dying leading to neurodegeneration.  The proteins needed for the proper function of synapses are osteopontin, platelet-derived growth factor (PDGF), fibroblast growth factor (FGF), and insulin-like growth factor (IGF). Also due to dysfunctional astrocytes, neuroligin, ephrin, and serine are all proteins needed for proper neuroplasticity leading to deficits in synapsis and the formation of microglia dysfunction. Microglia work alongside astrocytes and when astrocytes fail to function properly interleukin-6 (IL-6) and granulocyte-macrophage -colony-stimulating factor (GM-CSF) are upregulated to microglia. And in return microglia release interleukin 1-alpha (IL-1ɑ), complement C1, and tumor necrosis factor-alpha (TNF-ɑ). This feedforward mechanism perpetuates neurotoxicity. The authors have designed the figure based on the scientific information provided in references [22-31].

TDP-43 plays a major role in cellular signaling that activates an immune response leading to the mediation of neurological destruction and degeneration (Figure 2) [22]. It is especially notable in FTLD, ALS, and Alzheimer’s disease (AD) and is further researched in other neurodegenerative diseases [22]. Studies show that over-expression of TDP-43 activates glycogen synthase kinase-3β (GSK-3β) which is an important regulator of mitochondrial homeostasis including Ca^2+^ and phospholipid exchange [22]. TDP-43 and GSK-3β act as a second messenger for the activation of autophagy through autophagosome and lysosome fusion [22]. Recently, there has been a focus on analyzing lipid profiles in neurodegenerative diseases, such as ALS, and a focus on cardiolipin and its role in mitochondrial function in neuronal cells using Barth syndrome animal models [23]. Cardiolipin is a phospholipid component of the inner mitochondrial membrane and has a significant role in maintaining structure and integrity, mitochondrial homeostasis, and cellular signaling [24]. The translocation of cardiolipin from the inner mitochondrial membrane to the outer mitochondrial membrane plays an important function in mitophagy [24]. Lysophosphatidylcholine (LPC) and platelet-activating factor (PAF) are inflammatory lipids that induce cytokine production and activate caspase-1, a mediator for programmed cell death, in microglia [25]. In a study done by Phan et al. (2020) they found a marked decrease, almost 20%, of total cardiolipin in FTLD and an increase in LPC and PAF with subsequent increases of interleukin 6 (IL-6) and complement 3, two important proinflammatory cytokines [24,25]. There could be a homeostatic interaction between TDP-43, and GSK3b activation of lysosome fusion with cardiolipin for the exocytosis of the dysfunctional mitochondria [25]. Additionally, TDP-43 could have a connection with LPC and PAF in the induction of neurodegenerative inflammation [25]. This would be an area of further investigation for TDP-43 proteinopathies that have increased translocation and aggregation of TDP-43 to the mitochondria as seen in ALS, as well as its role in the signaling cascade activating gliosis (Figure 2). 

Glial cells with increased inclusions of TDP-43 have been shown to increase the inflammatory response [26]. Microglia, the phagocyte of the brain, are the first responders to brain injury and the first step in the neuroinflammatory cascade (Figure 2) [26]. It is thought that protein tyrosine phosphatase 1B (PTP1B) is an important regulator in cell signaling by activating nuclear factor kappa B (NF-kB). NF-kB is a group of transcription factors that is responsible for the DNA transcription of inflammatory cytokines [26]. Increased activation of TDP-43 has been shown to upregulate PTP1B expression, increasing cytokine production [26]. By inhibiting PTP1B, we can slow the neuroinflammatory response and early microglial signaling [27]. Studies are suggesting that people with certain gene mutations or variants in triggering receptor expressed on myeloid cells 2 (TREM2) and CD33, both highly expressed in microglia, have an increased risk of TDP-43 proteinopathies [27]. It is believed that TREM2 deficiency alters phagocytic clearance of TDP-43 by microglia which can account for the high inclusion density of glial cells in TDP-43 proteinopathies leading to enhanced neuronal damage [28]. This induces tumor necrosis factor-alpha (TNF-𝛼) and catalyzes necroptosis and destruction of the blood-brain barrier leading to the activation of astrocytes. Astrocytes with TDP-43 inclusions increase neuroinflammatory cytokines IL-1𝛽, IL-6, and TNF-𝛼 and when modulating these cytokines, it showed neuroprotective effects [29]. Recent studies show astrocytes also play a role in glutamine/glutamate metabolism [29]. Those with increased TDP-43 inclusions have depleted glutamine, the precursor for gamma-aminobutyric acid (GABA) responsible for the inhibitory pathway, leading to an unopposed excitatory pathway [29,30]. 

Another glial cell that is involved in gliosis of TDP-43 proteinopathy is oligodendrocytes (Figure 2). They are responsible for producing and maintaining myelin and axon stability [30]. Their increased iron and lipid metabolism allows oligodendrocytes to be more susceptible to oxidative stress from high-fat diets, amyloid-beta toxicity, TDP-43-aggregation toxicity, and the increased neuroinflammation that results from it (Figure 2) [30,31]. Oligodendrocyte progenitor cells (OPCs) are migratory cells that regulate and form new myelination after a demyelinating or neuronal-destructive event. They also form synapses with glutamatergic neurons in the hippocampus, cortex, and other parts of the brain seen in animal models [31]. The altered glutamate/glutamine metabolism seen in increased reactive astrocytosis could cause a disruption in the interaction of OPCs and glutamatergic neurons [31]. Without glutamatergic neurons, OPCs could fail to migrate and provide regeneration of neuronal tissue in the affected areas [31]. TDP-43 aggregation, loss of function, and toxicity have been large areas of focus in neurodegenerative diseases [31]. It activates and propagates the feed-forward glial neuroinflammatory cascade that allows for the rapid progression of neuronal damage, degeneration, and sclerosis [31]. Understanding the components and mediators of glial reactions will allow us to do further research in determining appropriate clinical solutions to either slow the progression of TDP-43 proteinopathies or prevent them from occurring.

Diseases associated with TDP-43 accumulation and toxicity

*Alzheimer’s Disease (AD)* 

AD is the leading cause of dementia and the main histopathological features are amyloid-beta (Aβ) and tau protein neurofibrillary triangles that lead to a variety of cognitive and neuropsychiatric symptoms [32,33]. The range of severity varies, but recent classifications of AD show immune destruction of TDP-43 inclusions is associated with the staging of AD progression [32,33]. TDP-43 inclusions in both amygdala and hippocampus have shown faster progression than with inclusions in just the amygdala or hippocampus or neither [32,33]. It is thought that there is an association between Aβ plaques and TDP-43 aggregation leading to activating microglia, reactive astrocytosis, and dysfunction of oligodendrocytes, but no cause has been established yet [34]. In familial AD, it is believed that gene variations of TREM2 and CD33, which are thought to be involved in microglial activation, lead to an increased risk of developing AD and worsening progression [34]. In a mouse model with overexpression of these two gene variations, it was shown that it increased neutrophil extravasation at the site of amyloid-beta plaques. Conversely, depleting neutrophils showed better outcomes of AD progression by decreasing Aβ plaque levels [34,35]. Another way Aβ plaque levels could be increased is through the role of lipid metabolism in astrocytes. A diet high in fats has been thought to increase Aβ deposition and increase the risk of developing AD [35]. Cholesterol indirectly suppresses the expression of ATP-binding-cassette transporter 1 (ABCA1) on astrocytes, which is important in the lipidation of apolipoprotein E (APOE). It does so by increasing amyloid-beta production through the β-site amyloid precursor protein (APP)-cleaving enzyme (BACE1) [35,36]. Furthermore, ceramide, a metabolite of fatty acid metabolism, has been shown to induce astrocytes to produce neuroinflammatory cytokines propagating the neuroinflammation-neurodegeneration cascade and beta-amyloid production [36]. Another player in gliosis is oligodendrocytes. They produce and maintain myelin and support axon stability, but are highly sensitive to oxidative stress [36]. Due to their sensitivity to Aβ and TDP-43 toxicity, this propagates the neuroinflammatory state and further damages neuronal tissue [36]. The overactivity of TDP-43 in relation to stress granule formation and ubiquitination caused by amyloid-beta proteolytic processing leads to mislocalization of TDP-43 from the nucleus to the cytoplasm and increased immune destruction/cell death resulting in terminal stages of loss-of-function/TDP-43 depletion, thus increasing transcription malfunctions of tau proteins and Futsch protein disrupting synaptic integrity [36].

*Parkinson’s Disease (PD)* 

PD is a neurodegenerative condition that is characterized by the loss of dopaminergic neurons in the substantia nigra and extending the basal ganglia [37]. It is a diagnosis based on clinical presentation and symptoms but also has atypical manifestations. This is still a large area of research and as a result, can be associated with a variety of phenotypes [37]. TDP-43 proteinopathies can appear as a range of phenotypes from ALS to FTLD and within that range there is an association of TDP-43 gene mutations that result in certain phenotypes of Parkinsonism, with the similar pathology of mislocalization and dysfunction of TDP-43 translocation back to the nucleus like in other neurodegenerative diseases [37]. The question of why an individual will express Parkinsonism rather than ALS or any of the other TDP-43 proteinopathies and phenotypes remains unclear [37]. The function of TDP-43 is localized in the nucleus to regulate gene transcription, RNA processing, and transport [37,38]. In its aberrant form, it becomes mislocalized and forms abnormal protein accumulation that ultimately leads to dysfunction in RNA metabolism and protein homeostasis, and neuron degeneration [37,38]. One of the TDP-43 targets is “Parkin'' a long intron pre-mRNA that is an E3 ubiquitin ligase involved in clearing damaged mitochondria by undergoing autophagy with the help of Phosphatase and tensin homolog (PTEN)-induced putative kinase 1 (PINK1) a serine/threonine kinase, to execute the mitochondrial quality control function [37,38]. This protein is normally transported into the mitochondria following translation to be cleaved and released into the cytosol for proteasome-mediated degradation [37,38]. The accumulation of TDP-43 proteinopathies causes damage to the mitochondria which does not allow PINK1 to be effectively cleaved and eventually becomes attached to the mitochondrial outer membrane [37,38]. Parkin is then recruited to mitochondria and induces mitophagy [37,38]. These have been found in early-onset Parkinson's disease. In a recent study, it was noted that TDP-43 selectively affects PINK1 protein turnover and causes cleaved PINK1 to accumulate in the cytoplasm due to the impairment of proteasomal degradation [37,38]. This leads to cytotoxic levels and lowers PINK1 levels and hence mitochondrial dysfunction and can possibly cause a gain of toxicity due to ectopic or increased phosphorylation of cytosolic substrate which ultimately leads to mitophagy and glial cell dysfunction [37,38].

*Limbic Predominant Age-Related TDP-43 Encephalopathy (LATE)* 

LATE is a TDP-43 proteinopathy of the limbic brain that mimics Alzheimer's-type amnesia dementia in persons over 80 years of age which is what distinguishes it from AD and FTLD [39]. It has similar pathology of less nuclear localizations of TDP-43 and increased cytoplasmic inclusions of neurons, astrocytes, and oligodendrocytes as well as the pathogenesis and involvement of these glial cells like in AD [39]. There’s a preference for localization to the medial parietal lobe, but can be seen in the amygdala, basal ganglia, hippocampus, and cortical structures with hippocampal sclerosis being described as the terminal point of severe progression like in other TDP-43 proteinopathies [39]. Recent observations have been made in analyzing genetic variations in LATE [39]. It was found that granulin precursor (GRN), Transmembrane protein 106B (TMEM106B), ATP Binding Cassette Subfamily C Member 9 (ABCC9), and APOE had significant gene association with pathology leading to LATE phenotypes and hippocampal sclerosis [39,40]. GRN, TMEM106B, and ABCC9 showed a change in expression when manipulating TDP-43 proteinopathy and APOE was shown to link AD to pathological changes [39,40]. results pave a new direction in future genetic research for all TDP-43 proteinopathies and the cause of toxic accumulation of TDP-43 that leads to destructive neuroinflammation and pathogenesis [39,40].

*Huntington’s Disease (HD)* 

HD is an autosomal dominant disorder caused by a trinucleotide, nucleobase coding triplet cytosine (C), adenine (A), guanine (G) (CAG), repeat mutation of the Huntingtin gene that is responsible for encoding the Huntingtin protein (HTT) and the eventual dysfunction of the subcortical motor circuits [41]. The mutant HTT, expanded polyglutamine, activates cellular stress response due to the abnormal protein production increasing stress granules (SG) [41]. Immunoreactivity to TDP-43 and SG-nucleating reticular activating system (Ras) GTPase-activating protein-binding protein 1 (G3BP1) inclusion stress granules were seen in the striatum as well as the superior prefrontal cortex, parietal cortex, and hippocampus [41]. The spreading of the pathology is suggested to be due to the effect played by extracellular vesicles involved in cell-cell signaling which includes miRNA that has a role in post-translational modification and possibly the formation of stress granules seen caused by unfolded HTT [41]. It is not the dysfunction of TDP43, but a rather conserved function of TDP-43 and fused in sarcoma (FUS), a DNA/RNA binding protein, that leads to the polyglutamine toxicity and TDP-43 aggregation seen in HD [42]. In the brains of Huntington patients, pTDP-43 aggregates are shown to co-localize with mutant Huntingtin (mHtt) inclusions [42]. Their expression of mHtt carrying 80-97 polyglutamine repeats in human cell cultures induces the aggregation and the phosphorylation of endogenous TDP-43 [42]. Mutant Htt aggregation precedes the accumulation of pTDP-43 and pTDP-43 co-localizes with mHtt inclusions [43]. The polyglutamine repeats are known to seed for TDP-43 aggregation in human cell culture. These aggregates ultimately damage the glial cells [43].

*Progressive Supranuclear Palsy (PSP)* 

PSP is a parkinsonism syndrome that has similar presentations to Parkinson's disease but cannot be diagnosed as such. PSP is characterized by the degeneration of the midbrain-diencephalic junction [44]. Studies have shown mechanistic links of spinal cord TDP-43 proteinopathies that are different from that observed in the brain [44]. Recently identified splicing factor proline/glutamine (SFPQ) in ALS pathogenesis and FUS interaction was altered in neurons containing TDP-43 aggregates in progressive supranuclear palsy, FTD, and ALS, and SFPQ expression were shown to be depleted in the same diseases, but not shown in AD, FTD, and globular glial tauopathy [44]. Moreover, TDP-43 aggregates had a positive correlation between microglial dysfunction in the anterior horn suggesting the pathogenesis of TDP-43 proteinopathy in the spinal cord and motor neuron dysfunction [44]. In a recent study, it showed a tauopathy in the classic pattern of progressive supranuclear palsy (PSP) in the form of neurofibrillary tangles were present in the entorhinal cortex and in the cornu Ammonis (CA1) section of the hippocampus and in the fusiform gyrus of the neocortex. These protein aggregates also accumulated in glial cells of white matter [44,45]. These inclusions are mainly found in cellular origin as tau-positive astrocytes, as either fibrillary or protoplasmic, coiled bodies, and glial threads [45]. This activates the glial cells and subsequently produces inflammatory markers that lead to cell death.

*Corticobasal Degeneration (CBD)* 

CBD is a rare condition characterized by worsening movement, memory loss, speech, and swallowing [46]. This condition is often overlapped with Progressive Supranuclear Palsy (PSP) due to the clinicopathologic and genetic similarities [46]. More often CBD is diagnosed as PSP when patients have downward gaze palsy [46]. The differentiation however is made during autopsy reports to determine whether it is PSP or CBD. In a study conducted observing TDP-43 levels in the brain, it was found that TDP-43 pathology was found in 45% of cases of CBD [46]. Then genetic analysis was performed and found that microtubule associated protein tau (MAPT) H1/H1 genotype frequency was significantly lower in TDP-severe (only 48% clusters observed) [46]. In another study conducted on CBD, TDP-43 immunoreactive inclusions were found in neurons and glial cytoplasm [47]. This illuminates the connection of TDP-43 impacting the glial cells and subsequently the neuronal cells resulting in neurodegeneration [47]. The mechanism in which this occurs has not been investigated [47]. However, TDP-43 aggregation and accumulation have been shown to lead to the dysfunction of the UPS in the cytoplasm [47]. Which is likely occurring in this disease process. Additionally, another proposed mechanism in which this occurs is through lysosomal dysfunction in the mitochondria due to the loss of TDP-43, resulting in accumulation and aggregation [47]. The formation of ROS and cytotoxicity leads to the death of the neuronal cells. This condition contributes to our list of neurodegenerative pathologies associated with TDP-43 proteinopathy [47]. However, the mechanism in which low levels or high levels of TDP-43 in the nucleus have not been studied to know which motif is affected [47]. Further investigation is needed at this time to determine which part of TDP-43 motifs is being affected [47].

*Neurofibromatosis Type 1 (NF1)* 

NF1 is a neurocutaneous congenital syndrome affecting organs of embryological ectoderm origin that includes the skin, eyes, and central nervous system [48]. This manifests in neurofibromatosis as multiple benign peripheral nerve sheath tumors arising from neural crest cells and affecting myelinated nerves [48]. It has an autosomal dominant inheritance or spontaneous mutation of the NF1 gene that encodes neurofibromin protein [48]. The loss of function of this tumor suppressor protein leads to an increase in RAS activity and cell proliferation that causes neurofibroma development and hyperplastic reactive gliosis [48]. Studies have shown increased glial fibrillary acid protein (GFAP) expression in post-mortem neurofibromatosis patients suggesting the idea of increased gliosis and proliferation of glial cells [48]. Neurofibromin also plays a role in maintaining synaptic integrity [48,49]. Neurofibromin and valosin-containing protein (VCP) interact to regulate the formation of dendritic spines [48,49]. There is data showing higher VCP and TDP-43 aggregation and decreased density of the dendritic spine [49,50]. The idea is that TDP-43 and VCP aggregation leads to a disruption of direct interaction of VCP and neurofibromin and thus decreased formation of dendritic spines [50]. Another study has shown that neuronal cells with mutated neurofibromin have an increased disruption of VCP and neurofibromin interaction leading to overexpression of VCP and VCP/TDP-43 aggregation, the decreasing density of dendritic spines, and disruption of synapse integrity [51].

*Lewy Body Dementia (LBD)* 

LBD is dementia that has histopathology of alpha-synuclein clumps called Lewy bodies (LB) [52]. It is split into two categories that are classified based on the onset of symptoms whether or not both cognitive and motor symptoms are presented within one year [52]. If cognitive symptoms appear greater than one year after the onset of motor symptoms, then it is considered LBD secondary to Parkinson’s [52]. The hallmark of this dementia with Lewy bodies is shown to have more of a neuropsychiatric phenotype compared to TDP-43 dementia which presents more of a neuromotor phenotype [52]. However, dementia with LBs can have TDP-43 comorbidity offering a wide variance of dementias, and LB and diffuse plaque density have interactions for total neuropsychiatric inventory (NPI-Q) in progress [52].

*Epilepsy and Other Pediatric Epilepsy Syndromes* 

These are chronic neurological disorders that are defined as multiple unprovoked seizures. There is a wide variety of possible causes of epilepsy including genetic, structural, metabolic, immune, and infections. There has been a recent focus on genetic manifestations, possible links between TDP-43 and glial cell activation, and their role in the metabolism of neurotransmitters [53]. In a recent study, the increased expression of TDP-43 in astrocytes induced a neuroinflammatory response in the hippocampus leading to sclerosis [53]. The increased reactive astrocytosis then depletes GABA precursor glutamine, altering inhibitory synaptic function and opening excitatory hippocampal circuits as outlined above [53]. In mice, models have shown protective and anticonvulsant effects when modulating pro-neuroinflammatory cytokines like IL-1β, IL-6, and TNF alpha [53]. This could be an area of genetic study for severe children epileptic syndromes like Lennox Gastaut syndrome and TDP-43-related pathogenesis of astrocytes.

*Trigeminal Neuralgia (TN)* 

TN is a disorder of the trigeminal cranial nerve (CN V) that is characterized by intense, sharp, pain in an area of the face when one or more of the three branches, the ophthalmic, maxillary, and mandibular, are affected [54]. In cases of primary trigeminal neuralgia, there have been reports of an association between TDP-43 inclusion and astrocytic gliosis in sensory and motor dysfunction of CN V [54]. Further studies show that increased expression of TDP-43 in astrocytes induces an increased neuroinflammatory response and could play a role in the progression of neurodegeneration [54]. Astrocytes also play a vital role in glutamine/glutamate metabolism, and increased activation of astrocytes depletes the GABA precursor glutamine altering inhibitory synaptic function [54]. No clear data has been shown, but it could explain the motor and sensory hypersensitivity seen in trigeminal neuralgia and further investigation would be needed.

*Fragile X* 

Fragile X is an X-linked dominant disease-causing mutation in the Fragile X mental retardation protein (FMRP) leading to neurological developmental delay, autistic-like behaviors, and focal seizures [55]. Recent studies have been focusing on TDP-43 and FMRP associations as they both share similar mRNA targets and work closely to maintain neuronal function [55]. One of the roles of TDP-43 is the downregulation of Futsch protein that is involved in maintaining synaptic integrity [55]. FMRP has a similar role and has different synaptic mRNA targets that regulate the translation of these synaptic proteins [55]. Co-aggregation of these two proteins leads to a cooperative repression mechanism and the loss of FMRP function is shown to increase the density of dendritic spines affecting neuronal synaptic plasticity [55,56]. FMRP expression is highly localized in the cytoplasm of microglia, astrocytes, and oligodendrocyte progenitor cells and seen in stress granules throughout the neuron, axons, and dendritic spines [57]. The dysfunctional FMRP leads to developmental delay due to dysfunctional neuronal synaptic plasticity. The mutated protein localizes in the cytoplasm and the co-aggregation mechanism seen with TDP-43 could account for the toxic accumulation of both proteins [57]. Over time the loss-of-function mechanism of TDP-43 like seen in other TDP-43 proteinopathies leads to the progressive neurodegeneration in Fragile X that is mediated by the feed-forward inflammatory cascade of microglia, astrocytes, and oligodendrocytes as outlined above [57].

*Attention Deficit Hyperactivity Disorder (ADHD)*****

ADHDis a neurological dysfunction disorder in which individuals exhibit inattention, hyperactivity, and impulsivity [58]. In a recent study in children with ADHD levels of TDP-43 and ubiquitin c-terminal hydrolase L-1 (UCH-L1) were evaluated [58]. The function of UCH-L1 is to remove misfolded proteins through the UPS. As previously stated, this pathway is important in the prevention of protein aggregation and accumulation [58]. In the study performed it was found that children with ADHD had higher levels of serum TDP-43 and UCH-L1 compared to healthy children [58]. The UPS contributes to the development of the central nervous system through the management of neuron function [58]. The high levels of UCH-L1 can be correlated to the higher levels of TDP-43 [58]. The more the UPS has to degrade misfolded proteins, the higher the level of UCH-L1 will be as they are correlated with one another [58]. The imperative point to see is that TDP-43 is elevated as well. The mechanism in which it is evaluated has not been evaluated [58]. However, in previous studies, it has been found that TDP-43 transgenic mice exhibit behavioral deficits, reduced social interaction, memory loss, and cognitive deficits [58]. This relates to ADHD as it is a behavioral deficit with a high level of TDP-43 expression [58]. In a similar format as other diseases mentioned, the dysfunction of the UPS due to higher levels of TDP-43 leads to toxicity in the UPS rendering it dysfunctional [58,59]. The dysfunctional UPS ensues in protein aggregation and accumulation, resulting in gliosis and subsequent neurodegeneration [58,59]. However, which level of overexpression of TDP-43 and gliosis is not known and is warranted for further evaluation [58,59]. It is important to note that the pathophysiology is similar to other neurodegenerative diseases and microglial activation theoretically occurs in ADHD as well [58,59]. The amount of gliosis is what needs to be studied. In the same study mentioned, IL-13 and IL-16 were at higher levels in symptomatic children with ADHD when compared to asymptomatic children on medication [58,59]. Higher levels of IL-13 were associated with higher levels of inattention while higher levels of IL-16 were associated with increased hyperactive-impulsive symptoms [58,59]. The two pro-inflammatory cytokines are found commonly in microglia [58,59]. When activated microglia release pro-inflammatory cytokines such as those mentioned and many more. Thus, in ADHD pro-inflammatory cytokines are released from microglia, especially for those who are symptomatic [58,59]. Microglia have been shown in other neurodegenerative diseases to potentiate inflammation in both the brain and the periphery [58,59]. More studies are warranted to evaluate the microglia's role in inflammation in ADHD and how much damage is being done [58,59]. A study conducted on this will elucidate the degree of damage when compared to other more aggressive neurological disorders, as ADHD has not been shown to have extensive damage to the brain like a disease such as Alzheimer’s [60]. However, ADHD and Alzheimer's according to the studies conducted are similar in the mechanism in which the cells are damaged through the UPS and glial cells. Determining the beginning point such as a gene for TDP-43 regulation would lead to a possible curative treatment.

*Schizophrenia* 

This is a highly heritable psychiatric disorder that features symptoms of psychosis (positive symptoms), blunting of normal affect (negative symptoms) and cognitive impairment [61]. The etiology of schizophrenia is still largely undiscovered, but it is believed to be related to dysregulation of dopaminergic activity in the mesolimbic and prefrontal cortical pathways leading to the positive and negative symptoms of schizophrenia [61]. Recent observations have been made of neuropsychiatric symptoms presenting in neurodegenerative diseases with studied TDP-43 associated dementias such as Alzheimer’s, FTLD, LATE-NC, and so forth that have affected limbic structures [61]. In FTLD mouse models and postmortem patients, disrupted in schizophrenia 1 (DISC1) has shown to aggregate with TDP-43 disrupting local dendritic translation resulting in decreased synaptic protein expression leading to the neuropsychiatric and behavioral deficits that were observed in these models [61]. Thus, TDP-43 and DISC1 aggregation lead to complex destruction of local translation in the dendrites of neurons in the limbic structures of the hippocampus, amygdala, and cingulate gyrus [61]. This could extend to astrocytes and its role in glutamine/glutamate metabolism that was outlined above as DISC1 has been found to regulate astrocytes through modulation of Ras-Raf-MEK-ERK pathway (RAS/MEK/ERK) second messenger signaling [61,62]. Early ubiquitination of DISC1, possibly through the TDP-43 ubiquitination pathway that leads to the coaggregation seen, alters gliogenesis in embryonic brain development that results in the dysregulation of GABA/glutamate [62,63]. Increasing the evidence that altering neurotransmission of the limbic structures by this mechanism can lead to the clinical features characterized by schizophrenia [63].

Diseases associated with TDP-43 depletion and/or loss of function

*Amyotrophic Lateral Sclerosis (ALS)* 

ALS is a fatal neurodegenerative disorder. Oftentimes when referencing ALS, FTLD is additionally mentioned due to the discovery of genetics and TDP-43 linkage in 2006 [64]. TDP-43 was a major component of the insoluble inclusions found in approximately 97% of ALS and approximately 50% in FTLD in post-mortem brains [64]. We will begin by discussing ALS first.

A mutation in TDP-43 leads to ALS and firmly establishes the causal role of TDP-43 for neurodegeneration [65]. In both familial and sporadic ALS TDP-43 proteins form insoluble intracellular aggregates in ALS patients. This was found in both wild-type and familial ALS. According to Yang et al. (2022) even a large fraction of familial ALS do not have TDP-43 still forms aggregates of the protein [65]. TDP-43 proteins are evident in motor neurons and oligodendrocytes [65]. The presence of TDP-43 proteinopathy in motor neurons and oligodendrocytes is believed to cause motor neuron degeneration by both losses of TDP-43 function and toxicity [65]. In a study conducted on mice, the low-level expression of TDP-43 in vitro showed that overexpression of TDP-43 induces liquid-liquid phase separation in the cytoplasm, then upon stress transform into permanent aggregates [66]. The aggregates then draw out TDP-43 from the nucleus and subsequently kill the cell [66]. This occurs because TDP-43 has two recognition motifs, a nuclear localization signal, and a nuclear export signal [66]. Normally TDP-43 is localized to the nucleus, however, it can be shuttled to the cytoplasm and back [66]. TDP-43 also has its own regulatory auto feedback system [66]. If there isn’t enough TDP-43 in the nucleus, it will upregulate to create more [66]. It can be proposed that when there is a low level of TDP-43 in the nucleus due to it being shuttled to the cytoplasm, upregulation of TDP-43 occurs [66]. This can become dysfunctional with TDP-43 mutations, shuttling more into the cytoplasm and creating more in the nucleus [67]. However, due to the dysfunction, the cell will continue to create more TDP-43 thinking the nucleus levels are low because it keeps getting shuttled out to the cytoplasm. Thus, more and more TDP-43 proteinopathy occurs [67]. In another study conducted, von Hippel-Lindau/Cullin-2 (VHL/CUL2) complex was shown to be responsible for ubiquitin ligase of misfolded TDP-43 fragments [67]. Overexpressed VHL resulting from misfolded TDP-43 and cytoplasmic inclusions in oligodendrocytes in mice leads to immunoreactivity suggesting the cause of oligodendrocyte dysfunction in ALS [67]. Additionally, ALS has been linked to the superoxide dismutase 1 gene (SOD1) mutation. The mutant SOD1 in oligodendrocytes leads to cellular dysfunction. Thus, the oligodendrocytes are not able to support neuronal axons [67]. In another study, TDP-43 knock-out mice were created and had progressive neurodegeneration to the extent that patients with ALS have [68,69]. This shows the connection between TDP-43 loss of function and its subsequent effect on motor neurons, which is degeneration through the loss of oligodendrocytes and other glial cells [68,69]. In one experiment, increased superoxide dismutase type 1 (SOD1) mutations activated inflammatory pathways specifically in astrocytes. The increased inflammatory cytokines released from astrocytes through the NF-𝜅B pathway are believed to have created toxicity in the neurons [69]. Further investigation in evidence through confirming higher serum/CSF levels of pro-inflammatory cytokines released from astrocytes through the NF-𝜅B pathway will confirm this mechanism in ALS [69].

Frontotemporal Lobe Dementia (FTLD)

FTLD is a neurodegenerative disease in which patients typically present with behavioral and personality changes subsequently followed by memory loss. This neurodegenerative disease has been linked to TDP-43 functionality and lack thereof [70]. In an experiment done by the Mayo Clinic, a marked reduction in UNC-13 homolog A (UNC13A) protein was found in TDP-43-depleted cells [70]. TDP-43 was additionally found biochemically in both the white and gray matter of the brain [71]. This finding is suggestive of not only neuronal pathology but also glial pathology due to the location of neuronal cell death [71]. As previously discussed, TDP-43 has its own feedback system where low levels of TDP-43 in the nucleus upregulate to create more [71]. The dysfunction of the TDP-43 in FTLD could be upregulating the nuclear export signal, moving TDP-43 from the nucleus to the cytoplasm [71]. TDP-43 has an autoregulatory feedback mechanism, thus when the nucleus observes low levels of TDP-43 it upregulates to bring the levels back to normal [71]. However, the nucleus continues to have low levels of TDP-43 because the nuclear export signal continuously shuttles TDP-43 out of the nucleus [71]. Thus, creating an endless cycle of TDP-43 aggregation and accumulation in the cytoplasm [71]. TDP-43 has been found in the inclusions of frontotemporal lobar dementia with genetic mutations in the progranulin gene (PGRN) and valosin-containing protein gene (VCP) [71]. In a study, it was suggested that PGRN is needed in order to keep lysosomes healthy [71]. The disruption of the lysosomes leads to the death of cells [71]. As previously stated TDP-43 is important in many pathways and proper function is imperative for the proper function of these pathways.

Therefore, in FTLD it is found that a low expression of TDP-43 affects the lysosomal function. Aggregation of TDP-43 in the cytoplasm causes an increase in work in the autophagosome-lysosome fusion [71]. The lysosome cannot keep up with the new workload of TDP-43 in the cytoplasm [71]. As the cell tries to recruit more lysosomes to maintain balance with the increased workload it fails. Thus, impairment in the autophagosome-lysosome fusion [71]. This process is similar to that of ALS and astrocytes. Astrocytes are innately neuroprotective, however, when overwhelmed become neurotoxic as proinflammatory cytokines are released at a higher rate [71]. Cell death occurs leading to neurotoxicity. In a study, chromosome 9 open reading frame 72 hexanucleotide repeat expansion (C9orf72 HRE) caused neuroinflammation in FTD [71]. It was observed that those carrying C9orf72 HRE markers exhibited FTLD symptoms earlier in life and TDP-43 pathological lesions [71]. It is suggested in the study that the mechanism in which neuroinflammation occurs is that of ALS, the NF-𝜅B pathway via astrocytes [71]. The activation of NF-𝜅B pathway is activated via astrocytes ensuing release of pro-inflammatory markers causing neuroinflammation and leading to the death of the neurons [71]. C9orf72 HRE was also found in microglia and when haploinsufficient knockout mice were observed, it was shown to have an increase in proinflammatory cytokines [71]. The increase in cytokines leads to neuroinflammation. These experiments confirm that down the line the pathway of TDP-43 proteinopathy causes neuroinflammation [71].

*Autism Spectrum Disorder (ASD)* 

ASD is a neurodevelopmental disorder with a spectrum of characteristics. The disorder ranges from behavioral and communicative deficits. In a preliminary study, TDP-43 levels were observed in children with and without autism [72]. Low levels of TDP-43 were observed in children with autism [72]. It is thought based on this study, that during early development low levels of TDP-43 may have contributed to the dysfunction of the ubiquitin-proteasome system [72]. As previously discussed, low levels of TDP-43 lead to dysfunction in the ubiquitin-proteasome system (UPS). TDP-43 is normally cleared through the ubiquitin-proteasome system within the cytoplasm [72]. This leads to increased levels of TDP-43 ubiquitination and due to the mislocalization, they then become ineffectively removed. Subsequently, this leads to the accumulation and aggregation of TDP-43 [72]. Therefore, it could be a contributing factor in disturbing neuroplasticity in autism. Additionally, the connection between TDP-43 and ASD is important to note as this could potentially be a TDP-43 proteinopathy with further research and observation in a larger study on ASD.

*Krabbe’s Disease (KD)* 

KD is a rare fatal neurodegenerative disease that due to the lack of galactosylceramidase there is extensive destruction to the myelin sheath surrounding the nerves in the brain [73]. The mitochondria function to produce myelin and oligodendrocytes [74]. In the review of the manuscript by Lucini and Braun, they discuss the role of mitochondria in TDP-43 proteinopathy [74,75]. In vivo and in vitro models an accumulation of TDP-43 in the mitochondria was observed [74,75]. This led to the destabilization of the mitochondria resulting in the formation of reactive oxygen species, which causes cytotoxicity resulting in apoptosis and necrosis of the cells [74,75]. Additionally, TDP-43 was found to have mitochondrial localization motifs [75]. In another study, the loss of expression in TDP-43 also decreased mitochondrial trafficking in both axons and dendrites [74,75]. As one of the functions of the mitochondria is to produce myelin, it is hypothesized to be the mediator of glial cell loss [75]. In Krabbe’s disease, the pathophysiology lies in myelin sheath destruction [75]. This area additionally needs to be investigated as the linkage of TDP-43 and cytokine elevation ultimately leads to neuroinflammation and subsequent neuronal death.

Niemann-Pick (NPC) Disease

NPC is a rare lysosomal storage disorder that specifically affects the body’s ability to metabolize fat within cells [76]. Over time this can affect many organs, most importantly for our research, the brain [76]. In Niemann-Pick disease there is neurodegeneration. The mechanism by which the brain is affected is unknown [76]. In one study the expression and localization of TDP-43 in mice and human models of NPC disease were performed [76]. In this work, it was found to have an abnormal expression of TDP-43 in the cytoplasm of the cerebellum of mice and in human in vitro models with NPC [76]. After the loss of function of TDP-43 within the cytoplasm stress granules were appreciated [76]. It is hypothesized that alteration in the localization of TDP-43 may contribute to neurodegeneration pathogenesis [76]. As previously discussed TDP-43 is localized in the nucleus and when it is mislocalized to the cytoplasm at higher levels due to the dysfunction of the nuclear export signal, the ubiquitin-proteasome system cannot handle the overwhelming accumulation and aggregation, which lead to neuronal apoptosis [76]. This will release toxins that are neutralized by astrocytes and microglia, which indeed release their proinflammatory markers that result in neuroinflammation and apoptosis of neurons [76]. This pathway is continuously seen in neurodegeneration.

*Tuberous Sclerosis Complex (TSC)* 

TSC is a genetic disorder characterized by tumors or hamartoma formation in multiple organs [77]. When there becomes neurological involvement it presents as epilepsy, intellectual disability, and autism [77]. Tuberous sclerosis is caused by mutations in TSC 1 or TSC 2 genes [77]. Mutated TSC genes lead to hyperactivation of the mammalian target of the rapamycin complex 1 (mTORC1) pathway [77]. In an experiment performed on mice with TSC, an elevation of cytokines was observed. Most notably IL-1β because activation predominantly occurs in astrocytes and leads to proinflammatory cytokines being released [77]. In another study, it was hypothesized that astrocytes are the glial cells that are being affected by TSC [78]. It was observed that TSC astrocytes showed an increase in proliferation and changes to gene expression than the control astrocytes [78]. This is significant because of the roles which astrocytes play in the neurological system. The main function is for neuroprotection, however, in the event they become dysfunctional they become neurotoxic [78]. In the same experiment, it was hypothesized that aberrant mTORC activation causes increased proliferation of TSC cells [78,79]. This was confirmed through mTORC inhibition and subsequent restoration of TSC cells [78,79]. This is important because another experiment confirms that mTORC1 is a molecular target of TDP-43 through direct interaction [79]. In the study, it was found that TDP-43 depletion causes instability of mTORC lysosomal localization and mTORC1 activity [79]. The depletion and reduction of lysosomal autophagosomes lead to neurotoxicity in animal experiments [79]. Although at this time TSC has not been linked to TDP-43 directly, these experiments have shown the need for further investigation to confirm TDP-43 levels [79]. Based on the information TDP-43 is likely connected to TSC through mTORC1 [79]. A low level of TDP-43 or a deficiency leads to a reduction and instability in mTORC1, mTORC1 is unable to be activated leading to an impaired autophagosome-lysosome fusion [80]. It is hypothesized by our researchers that a low level of TDP-43 causes a significant reduction in mTORC1. The reduction in mTORC1 leads to an impaired autophagosome-lysosome fusion as previously stated, ultimately leading to protein aggregation and accumulation [80]. Thus, this is why astrocytes are being affected in TSC disease and an elevation of proinflammatory cytokines is observed.

Diseases associated with no changes in the level of TDP-43

There is an increasing amount of research and evidence supporting the significance of TDP-43 and its role in neuronal cells. It is important to outline the normal function of TDP-43 and how it contributes to neurological homeostasis and neuronal remodeling, or gliosis. In these diseases, there is a lack of evidence of TDP-43 involvement in the pathogenesis or progression of the illness. Even though data has not shown a change in levels or an association in toxicity of TDP-43, it is not implausible to believe that TDP-43 contributes to the activation of glial cells and neural cell destruction through one of its many mechanisms that are continuously being studied. Understanding the similar pathophysiology of gliosis and neuro destruction in TDP-43 proteinopathies and non-TDP-43 proteinopathies we can compare and contrast and hopefully provide a new direction in the future research of neurodegenerative diseases.

*Guillain-Barre Syndrome (GBS)* 

GBS is an acute, post-infectious, inflammatory, and demyelinating polyneuropathy caused by cross-linking the body’s autoantibodies to its own axonal antigens [81]. This condition is a rapidly progressive neuroinflammatory disease characterized by symmetric and ascending flaccid paralysis [81]. The most common cause of GBS is infection by campylobacter enteritis that mediates inflammatory response, which typically presents several weeks after the gastrointestinal infection [81]. This triggers the humoral response and several reports have shown a cross-reaction of autoantibodies to gangliosides of peripheral Schwann cells, which is a type of glial cell responsible for the myelination of peripheral motor and sensory neurons [81]. The peripheral nervous system has an increased ability for axonal regeneration compared to the central nervous system [81]. Schwann cells switch to a growing state rather than a conducting state in response to axonal damage [81,82]. Cellular debris from Schwann cells stimulates toll-like receptors (TLR), to recruit and activate macrophages to prepare axons for regeneration [81,82]. The first half of debris clearance is done through autophagy and the rest is done by phagocytic macrophages via chemotaxis by cytokines and myelin degradation products from Schwann cell processing [81,82]. The slowing of signal conduction along the axon due to the loss of myelin causes muscle weakness and the extent of degeneration is related to the severity of the immune response [81,82]. Due to its acute onset caused by mostly infectious etiology, the aberrant TDP-43 mechanism in pathogenesis is not a component of this disease [81,82]. However, evidence is continuously being gathered for TDP-43 function as a cell-to-cell signaler and its role in autophagy and cell death [81,82]. In peripheral cells, it has been observed that TDP-43 increases the amount of naturally occurring antibodies (nAbs), especially immunoglobulin (IgG4), in ALS phenotypes that have increased TDP-43 aggregation [83]. It is possible that TDP-43 is a component in the formation of antigens that react to autoantibodies and would need to be expanded on more. Future research can also explore how TDP-43 functions in peripheral neurons and compare the roles seen in the central nervous system. It is likely that it is a mediator in the autophagy and degradation of myelin in Schwann cells as observed in oligodendrocytes of the CNS.

*Creutzfeldt-Jakob Disease (CJD)* 

CJD is the most common human neurodegenerative prion disease and it is caused by the sporadic, familial, or infectious conversion of normal prion protein (PrPC) to conformationally misfolded and altered prion protein scrapie (PrPSC) [84]. Normal PrPC contains an alpha-helical structure whereas abnormal PrPSC has more beta pleated sheets, which leads to accumulation and plaque formation resulting in cell toxicity and death [84]. Similar presentations of the pathogenesis of amyloid-beta plaques with TDP-43 accumulation and PrPSC accumulation in neurons have been observed and have prompted researchers to check for an association [84]. However, through the use of immunolabeling, TDP-43 cytoplasmic accumulation was not observed in the ubiquitination of PrPSC plaques and would be considered separate neurodegenerative categories [84]. Normal PrPC plays a variety of crucial functions similar to that of TDP-43 even though it’s not associated. These roles include copper metabolism in response to oxidative stress, immune function and upregulation of T-cells, regulation of apoptosis and autophagy, and ubiquitination [85]. PrPSC neurodegeneration is induced by glial cells through similar accumulation mechanisms seen in TDP-43 neurodegeneration and is believed to be the cause of infectious seeding throughout the brain [85]. Observations of PrPSC have been shown to impact astrocytes and microglia, but not oligodendrocytes [85]. Schwann cells, however, are affected but aren’t involved in the neuroinflammatory cascade. It’s thought that prions are introduced in the periphery and transported to the CNS [85]. It is then further spread throughout the neuronal circuit via axonal transport [86]. Affected Schwann cells could offer the entry point that allows it to reach the CNS and the unaffected oligodendrocytes maintain the axonal and synaptic integrity needed for this pathogenesis [86]. It has been proposed that astrocytes are independently infected and responsible for the release of neurotoxic PrPSC, while microglia clear the protein and activate the inflammatory cascade. Additionally, astrocytes are not affected by the toxicity, but the neurons near the astrocyte culprit are, thus further promoting the astrocytic role in the spread of infection in the CNS.

*Neurosyphilis Tabes Dorsalis (NTD)* 

NTD is a result of untreated syphilis and subsequently causes a slow degeneration of the dorsal root and dorsal columns [87]. Years after primary infection it was seen that chronic inflammation with subsequent Aβ deposition, tau phosphorylation, and degeneration was observed [87]. In a study conducted by Danics et al. (2021) they observed the brain of 11 patients with neurosyphilis and all had severe gliosis. Additionally, tau-containing astrocytes were observed. The study showing gliosis and Aβ deposition are imperative to further research as the study was limited to 11 patients [87]. Additionally, the connection with AD is important to note as this could potentially be a TDP-43 proteinopathy with further research and observation in a larger study in neurosyphilis [87]. The infection causes chronic inflammation that leads to proinflammatory cytokines being released. The cytokines then damage the neurons and certain parts of the dorsal root leading to tabes dorsalis, the tertiary stage of syphilis [87]. In a recent study, neurosyphilis was associated with rod-shaped microglia [88]. In other neurodegenerative diseases, rod-shaped microglia are present as well, including Alzheimer’s and Lewy body disease [88]. Rod-shaped microglia are found wrapped around degenerating apical dendrites in upper motor neurons [88]. TDP-43 pathology in astrocytes was also observed in the phosphorylated neurofilament tangles [88]. This shows a connection between neuroinflammation and TDP-43 [88]. The levels were not investigated in this study, however, TDP-43 pathology is involved in the mechanism in which syphilis causes neurodegeneration of the motor neurons. One hypothesis is that the infection makes its way to the brain causing inflammation of the neurons, as astrocytes and microglial cells try to clean up the infection they become overwhelmed [88]. As the cells try to degrade cells through autophagosome lysosome degradation the cell continues to increase TDP-43 levels, which is likely why we see TDP-43 pathology in astrocytes and in neurofilaments in this disease. To affirm or refute this hypothesis TDP-43 levels are to be investigated [88].

*Herpes Simplex Virus-1 (HSV-1)* 

HSV-1 is a virus that commonly causes cold sores. The virus sits dormant in the dorsal root ganglia and occasionally reactivates under stress. HSV-1 occasionally spreads to the central nervous system causing viral encephalitis [89]. In recent data, HSV-1 has shown a possible connection to AD, specifically sporadic AD. HSV-1 has been shown to damage neurons and glial cells, through inflammation and subsequent alpha-beta deposition and tau phosphorylation [89]. In regions of the inflammation found in the brain from HSV-1, it was observed to have phosphorylated tau immunopositively neurons in the medial temporal lobe [89]. This pathology is also associated with AD neuropathological changes. Due to limited cases in the study, no TPD-43 protein was observed. However, HSV-1 encephalitis patient’s Aβ deposition [89-91]. This is important to note because in Alzheimer's disease Aβ deposition and TDP-43 inclusions are observed with one another. HSV-1 is presented similarly, and thus further investigations are to be performed. The responders to infections such as HSV-1 in the brain are microglia and astrocytes [89-91]. Microglia is notably the first to respond to HSV-1 [89-91]. Oligodendrocytes following an HSV-1 infection can secrete microvesicles containing viral particles resulting in cell death of neurons, cell death, and demyelination [89-91]. The host activates pattern recognition receptors (PRRs) for effective antiviral response during HSV-1 brain infection, which primarily engages type I interferons (IFNs). As a consequence of HSV-1 infection, astrocytes undergo “reactive astrocytosis”, which involves morphological and molecular changes that result in increased astrocyte proliferation, change of cell morphology with a loss of astrocytic projections, and increased levels of proteins, such as glial fibrillary acid protein (GFAP) [89-91]. HSV-1 up-regulates astrocytic secretion of TNFα and IL-6 and induces the production of type I IFNs through the activation of the NF-𝜅B pathway [89-91]. In another study HSV-1, infected astrocytes were activated and shown to express proinflammatory neurotoxic markers [89-91]. In one of the markers, the HSV-1 infected cell protein triggered fibroblast growth factors (FGFs) activity [89-91]. Through paracrine neurotrophic signaling spread to other cells. Those inflammatory changes lead to apoptosis of glial cells and neurons [89-91]. Although in these specific studies aforementioned TDP-43 was not observed, it is likely they are contributing.

Kennedy Disease (Spinal and Bulbar Muscular Atrophy)

Kennedy disease, reclassified as a neuromuscular disease, is an X-linked mutation of misfolded androgen receptor (AR) polyglutamine (PolyQ) accumulation in the muscles and motor neurons in the spinal tract and dorsal root ganglions [92]. Animal models show direct pathogenesis due to toxicity of ARPolyQ aggregation in the muscle rather than the motor neurons since skeletal muscles are a direct anabolic androgen receptor [92]. The sensitization of AR is caused by testosterone-induced misfolded ARpolyQ conformations to the nucleus where cellular dysfunction takes place [93]. However, one way to prevent translocation of misfolded ARPolyQ to the nucleus is through autophagy which is blocked by the accumulation of the protein, which is similarly shown in ALS via TDP-43 aggregation [93]. Inducing transcription factor EB (TFEB), autophagy master regulatory, with trehalose has shown to increase autophagic flux and clearance of cytoplasmic aggregates in neuronal models [93-95].

Diseases associated with no check of the level of TDP-43, but glial cells involved

There are different mechanisms involved in multiple diseases illustrated below. Glial cells are involved either directly or indirectly in the pathogenesis of these disorders, while TDP-43 has not been investigated yet. With the advanced technologies, we were able to understand the exact pathophysiology of each disease, this also should enlighten the scientists to further investigate TDP-43 involvement given its function within the neurons. Since the glial cells contributed to the pathology of such disorders, TDP-43 already has clear communication with those cells within the neurons whether initiating activation or inactivating glial cells. We highly suggest extensive investigation of TDP-43 as a contributor to the following diseases.

*Aicardi Goutieres Syndrome (AGS)* 

AGS is a genetic interferonopathy disorder that affects the brain, spinal cord, and immune system [96]. This disease leads to progressive neurologic deterioration and severe developmental delay, as well as early death. The average lifespan of an individual with AGS is less than five years of age [96]. In the studies conducted thus far, the pathophysiology of AGS remains unknown, however, there are a few proposed mechanisms. One proposed mechanism is believed to be an accumulation of nucleic acids which triggers an inflammatory immune system [96]. The inflammatory immune system response ends up damaging the myelin sheath. In fact, in all cases of AGS, the common biomarker that is found is interferon-alpha (IFN-𝛼) [96]. IFN-𝛼 is an inflammatory marker causing damage to the myelin sheath. The causative agent that produces the IFN-𝛼 is believed to be the astrocytes, as they produce IFN-𝛼 and its subsequent response, inflammation [96]. The damage from inflammation leads to death of the myelin sheath and in turn neurodegeneration is observed. One important factor of this mechanism that remains unanswered, is what causes the astrocytes to produce IFN-𝛼 [96]. The function of the astrocyte in the brain is to provide energy to the neurons, maintain the blood-brain barrier, and aid in excitatory neurotransmission. As previously stated, the function of astrocytes is seen in the excitatory pathway [96]. A possible mechanism can be protein tyrosine phosphatase 1B (PTP1B) inhibition. In one study, it was found that PTP1B expression in astrocytes was upregulated by TDP-43 overexpression [96]. PT1P1B was shown to activate NF-𝜅B, which led to the production of proinflammatory markers by subsequent astrocytes. Based on these findings, AGS is likely linked to the mechanism of TDP-43 overexpression and further studies are warranted to investigate this very possible pathway.

*Wilson’s Disease (WD) and Menkes Disease (MD)*****

WD and MD share similar pathophysiology**.** The glial cells, specifically astrocytes, are key regulators of extracellular ion hemostasis of redox-active metal iron and copper in the brain [97]. These cells are equipped with Menkes protein ATP7A/B a copper-transporting P-type ATPase that undergoes reversible copper-dependent transport between the trans-Golgi network and vesicular structures [97]. They have the ability to take up, store, and export copper as needed to maintain a delicate balance within the brain. Disturbances in copper homeostasis occur in both Menkes and Wilson's disease. For example, increasing copper levels can have a negative impact on the brain [97]. Wilson's disease, due to mutation in Menkes protein, causes excessive uptake in copper. The accumulation of redox-active copper form and the disruption of hemostasis catalyzes the production of hydroxyl radicals by the Fenton-like reaction [97]. The glial cells, neurons, and ultimately brain induce an oxidative stress state, damaging all cells. The oxidative stress and cell damage contribute to the neurological phenotypes found in Wilson's disease. In Menkes disease, it's the reverse effect, with a copper deficiency state [97,98]. Glial cells will take up the majority of copper but fail to export it to other cells of the brain and thus, leaving the environment in a deficient state [97,98]. The mineral accumulates in astrocytes and stimulates hydroxyl radicals which damage astrocytes themselves. The brain and neurons suffer from a non-homeostatic balance and are not able to survive without copper present as it acts as a cofactor for many physiological processes in the brain [97,98]. An intriguing question remains unsolved, that is, what cell-to-cell interactions occur during the physiological response mechanism to the oxidative state? Multiple studies have proposed the possibility of an IFN activation leading to enhanced inflammation by T-helper (TH2) immune cells. We highly suggest investigating the exact mechanisms of microglial-immune cell interactions.

*Neuronal Ceroid Lipofuscinoses (NCLS)* 

NCLS, also known as Batten disease, is an inherited neurodegenerative disease that affects children [99]. TDP-43's role in this disorder is not researched, however, glial cells are and have been found not only in disease progression but the prediction of neuronal loss location [99]. In a study conducted by Parviainen et al. (2017) in mouse models as well as human autopsy material, it was found that glial cell response and morphological transformation were less pronounced in astrocytes and microglia cells [99]. Both cell types play key roles in neuron function and survival, therefore any compromise leads to dysfunction in them. Very little is known about how mutations in genes lead to disease phenotypes in the brain. Glial activation of both astrocytes and microglial cells is known to occur early throughout the progression of the disease. The reason for its early activation still remains unsolved. However, we do know their response is attenuated and the morphological transformation is delayed [99]. The inherited disorder causes mutations in specific genes that prevent it from excreting cellular waste. These include proteins and lipids which accumulate in cells as aggregates. Aggregate buildup disrupts the actin/intermediate filament cytoskeleton, calcium signaling propagation, and glutamate clearance [99]. Therefore, cells can't morphologically change and their activation is delayed. Secretion of neuroprotective factors such as chemokine, cytokines, and antioxidant properties via glutathione are dramatically reduced. The dysfunction in glial cells triggers neurodegeneration in the CNS and exacerbates the clinical findings present in this disease [99,100]. We highly suggest more investigation into what cytokines and chemokines are involved in this mechanism. As mentioned above, IFN is proposed, however, the complexity of glial cells seems to favor a multifactorial reaction with various markers presents contributing to the inflammation.

Dandy-Walker Syndrome (DWS)

DWS is a cerebellar malformation that is characterized by cerebellar hypoplasia, an enlarged posterior fossa, and dilated fourth ventricle [101]. It is believed to be caused by gain of function mutations that cause heterozygous deletions of the zinc finger protein of cerebellar 1 (ZIC1) and zinc finger protein of cerebellar 4 (ZIC4) on chromosome 3q25.1 [101].

These individuals express phenotypes that cause craniosynostosis of coronal sutures, with variable learning disabilities [101]. Even though TDP-43 correlation with Dandy-Walker disease has not been researched, effects in glial cells have, and can be seen affecting pathogenesis. In a recent study, the disease was shown to be associated with a mutation that causes loss of function of the forkhead box C1 (Foxc1) gene, a transcription factor protein that acts in the cerebellum to induce radial glial mitosis and a chemoattractant for nascent Purkinje cells [101]. The receptors for Foxc1 protein (Cxcr4) are expressed in glial cells [101]. Loss of the transcription factor reduces the proliferation of cerebellar ventricular zone radial glial cells and embryonic neuronal differentiation [101]. Morphological changes fail and thus migration fails. These particular cells are known as progenitor astrocytes that act as scaffolds for other nervous system cells to migrate across. Foxc1 deficiency leads to the loss of glial cells and disrupts the development of the cerebellum [101]. Disruption in mitosis and migration is normally seen in early embryonic brain development and exacerbates the neurodevelopment issues seen in Dandy-Walker syndrome. Much is not known about the exact pathway between Foxc1 and glial cells. We highly suggest further investigation of how Foxc1 interacts with glial cells and the specific signaling pathway required.

*Zellweger Spectrum Disorders (ZSD)* 

ZSD are a rare group of autosomal recessive disorders characterized by mutations in one of 13 peroxisomal biogenesis factors (PEX) genes [102]. These defects in peroxisome formation cause dysfunction of many metabolic pathways. It is a lethal condition that involves mutations in the PEX1 gene [102]. The primary function of the gene is to encode a set of proteins that form and maintain peroxisomes. The peroxisomes are commonly found in oligodendrocytes and Schwann cells, the major producers of myelin within the CNS and peripheral nervous system (PNS) [102]. They have multiple functions in peroxisome metabolism which include the following: fatty acids breakdown, peroxide detoxification, oxidizing amino acids and polyamines, and synthesis of bile acids and plasmalogens [102]. The membranes of myelin have a high lipid-to-protein ratio with lipids accounting for almost 70% of the membrane [102]. Another major component of the membrane is plasmalogen. These components are required for the integrity and stability of glial cells to undergo myelination and function normally. In Zellweger’s syndrome, demyelination occurs and production of myelin is reduced due to the lipid-rich make-up of myelin. The PEX1 mutation does not allow for the biogenesis of peroxisomes leading to complete or partial dysfunction [103]. As a result, the metabolism of alpha and beta-oxidation of fatty acids does not occur. Fatty acid accumulates within the cytoplasm of the cells and leads to deficiency in plasmalogen and hypomyelination of the CNS by glial cells. As a result, the CNS phenotypic abnormalities in Zellweger are expressed [103]. The mechanism of glial cells in Zellweger should be further investigated to find therapeutic drugs that will target the demyelination process.

*Friedreich’s Ataxia (FRDA)* 

FRDA is a genetic neurodegenerative disorder that is commonly associated with cardiomyopathy [104]. Neurodegenerative manifestations can arise early in the course and present with unsteady posture, frequent falls, and difficulty walking. Some individuals can develop ataxia, loss of speech, and scoliosis [104]. The disease is caused by mutations in the FXN gene that codes for a mitochondrial protein called frataxin [104]. The primary role of this protein is to regulate iron hemostasis and iron-sulfur cluster production. Glial cell mechanisms have been well understood in this disease and multiple pathways take place, leaving a detrimental impact. There is mitochondrial disruption, active iron form accumulation due to the Fenton and Haber−Weiss reactions, and production of hydroxyl radicals that ultimately exacerbate glial cell loss [104]. The primary producers of ROS within the CNS are microglial cells via the enzyme nicotinamide adenine dinucleotide phosphate (NADPH) oxidase 2 (NOX2) [104]. During an inflammatory response, there is an equilibrium between antioxidant defense and ROS production. However, equilibrium is disrupted in FRDA, glial cells release a continuation of reactive species causing a constant oxidative stress state and exacerbating the neurological phenotypes seen [104]. In addition, the iron-sulfur (Fe-S) clusters are involved in the electron transport chain within the mitochondrial [104]. Downregulation of frataxin causes mitochondrial dysfunction which subsequently reduces ATP levels and induces oxidative stress with ROS accumulating and consequently cell death [104]. The various aberrant pathways contribute to the phenotypic expressions seen in FRDA and the severity of the disease course is largely due to the glial cell abnormalities. Further investigation should be done to detect the exact mechanism and signaling as to how glial cells respond to ROS.

*Sphingolipidoses: Farber's, Fabry’s, and Tay-Sachs Disease* 

These diseases share similar pathophysiology. The plasma membrane is composed of many molecular components. One major component is the sphingolipids which form tightly packed membranes in myelin [105]. They have multiple roles that include providing structural support to glial cell membranes, myelination, and maintenance of myelin integrity as well as regulating the growth rate, differentiation, and CNS death [105]. The development and maintenance of the CNS require a homeostatic balance of membrane sphingolipids in both glial cells and myelin [105]. Any abnormality or change of this balance will lead to negative impacts on glial cells and therefore the CNS [105]. There are conditions that affect the sphingolipid metabolism and lead to disruption of this balance. These conditions are known as sphingolipidoses which are caused by specific enzyme deficiencies in sphingolipid metabolism in the lysosome and lead to demyelination in the CNS [106]. Three particular diseases, known as Farber's, Fabry’s, and Tay-Sachs disease have shown that glial cells play important roles in the pathogenesis of the three. In Farber’s disease, the deficiency is in acid ceramidase, an enzyme that converts ceramide to sphingosine. In Fabry’s disease, alpha-galactosidase is the deficient enzyme and Tay-Sachs has the deficiency in the β-hexosaminidase (HEXA) gene [106]. The lack of these enzymes causes the accumulation of sphingolipids as they cannot be hydrolyzed and therefore accumulate in the lysosomes with ceramide, trihexosylceramide, and gangliosides respectively [107]. The sphingolipids in glial cells accumulate in the lysosomes and inflammation takes place. Glial cells are activated and release inflammatory mediators IL-1 cytokine and receptors, IL-6, IFN, and TNF-𝛼. As a result, reactive oxygen species are produced and damage all cells within the brain resulting in demyelination. It is also known that the downward pathway of sphingolipid metabolism involves producing sphingosine-1-phosphate (SP1) [107]. The molecule interacts directly and signals with neurotrophin-3 and platelet-derived growth factors during the growth and myelination state of oligodendrocytes [108]. In addition, microglial cells produce SP1 during the first steps of myelin repair to allow for the migration of oligodendrocytes [108]. Downward metabolism does not occur and SP1 is not produced when the deficiencies are present. No repair or oligodendrocyte maturation occurs. The CNS stops myelination and brain development degenerates [108].

*Tourette Syndrome (TS)* 

TS is a neuropsychiatric disorder in which patients have a persistent tic, either motor and/or vocal [109]. The pathophysiology of TS is not well understood. However, we do know that the disease has a genetic and environmental predisposition [109]. New evidence via animal studies has shed light on a multifactorial process with glial cell abnormalities being the root cause. These processes include the following: the inability of glial cells to induce inflammation or have a protective effect on the brain, lack of survival support, and lastly abnormalities in synaptic pruning [109]. Microglial cells are the most important immune cells residing in the brain and have been classically associated with the induction of inflammation, neuron death, and degeneration of the CNS [110]. It is clear that glial cells have a neuroprotective and neurodegeneration role. Their activation is induced via TH2 cytokines and IL-4, in return, glial cells express insulin-like growth factor-1 (IGF-1) as a result and produce this neuroprotective phenotype [110]. IGF-1 is deficient in microglial cells of TS which causes a non-protective state and leaves the CNS susceptible to inflammation from environmental sources [110]. The glial cells fail to morphological change and inflammatory cytokines cannot initiate an immune response. Another cause is the pathological changes in the circuitry of the corticobasal ganglia [110,111]. New evidence from animal studies demonstrated that microglial cells can engulf additional synapsis during synaptic pruning, a normal process that happens in the CNS in postnatal development between neurons and microglial interactions through the fractalkine chemokine receptor [112,113]. The formation of the brain circuitry and normal connection requires microglial cells to undergo synaptic pruning [113]. It is well documented that fractalkine chemokine receptors do not function appropriately in TS and cause disruption to the signaling pathway between neurons and microglial cells [113,114]. The result of the disruption leads to various neuronal and behavioral dysfunctions commonly seen in TS. We highly suggest further investigation as to how the deficiency in the receptor diminishes synaptic pruning.

*Pseudotumor Cerebri (PC)* 

PC is a neurological condition presenting with increased intracranial pressure. Most believe it's a disease of CSF disturbance and venous hypertension [115]. However, new studies have demonstrated that the actual pathophysiological process occurs in the glial-neuro-vascular interface within the brain. This anatomical site is composed of glial cells especially astrocytes, neurons, capillaries, and pericytes [116]. The astrocytes provide foot processes in a circularly shaped manner to encase the basement membrane. They are a bridge between brain capillaries and neurons to regulate metabolism for fluid homeostasis [116]. The pathophysiology of intracranial hypertension (ICH) involves patchy astrogliosis that changes the configuration of astrocytes and neurons [116]. The blood-brain barrier eventually is disrupted and leaks blood fibrinogen and fibrin [117]. Astrocytes become activated, release aquaporin-4 (AQP4), and inflammatory response by producing cytokines, including IL-1β, IL-8, and TNF-α [117]. The changes in the blood brain barrier alter intracranial pressure volume reserve which will increase volume and therefore intracranial pressure.

*Central Pontine Myelinolysis (CPM)* 

CPM is characterized by the non-inflammatory demyelination process of the basis pons [118]. There are multiple causes of this condition, however, one, in particular, seems to be the main culprit. That is the rapid correction of chronic hyponatremia. It is also known to occur as a complication of severely ill conditions which alter Na+ levels. The demyelination process affects all cell types including the loss of oligodendrocytes [118]. The changes that occur in serum osmolarity and fluid distribution lead to cell fluid loss. A key player in this disease is known as the astrocytes which have been demonstrated to have important roles in the pathogenesis [118]. They are abundant in the CNS relative to other neurons and encase the brain vasculature and synapses [119]. They are involved in controlling the blood-brain barrier. They are known to maintain hemostasis and regulate any changes in brain fluid volume using aquaporin channels. Astrocytes have genes that express aquaporins (AQP1 and AQP4) [119]. Any disruption to astrocytes will potentially lead to no or reduced expression of AQP1 and AQP4 and loss of volume homeostasis within the brain [119]. The demyelination of astrocytes following rapid correction causes apoptosis and subsequently reduced aquaporin expression in astrocytes to regulate the fluid balance [119]. As a result, the astrocytes exacerbate the condition in the central pons.

*Wernicke-Korsakoff (WK) Syndrome* 

WK syndrome is a neurological structural disease that damages certain brain regions. Patients with WK syndrome have neuropathological changes in the medial thalamus, mammillary bodies, and cerebral cortex [120]. The main culprit is due to thiamine deficiency. The lack of thiamine can be caused by many things, typically alcohol, dietary or eating disorders, or chemotherapy [120]. There are three major processes that have been tested to be involved in this disorder as well [120]. Glial cells have been found to contribute to WK and explain cerebral cortex degeneration. Firstly, the loss of glutamate transporters expressed by glial cells. Secondly, the oxidative stress induced by losing glutamate transporters [120]. Thirdly, the change in the functional integrity of astrocytes. Astrocytes have many functions within the brain, a major role is their ability to maintain the balance of the neurotransmitter glutamate within the interstitial space [120,121]. They do this by expressing two glutamate transporter subtypes, excitatory amino acid transporters 1 and 2 (EAAT1/EAAT2) that can clear neurotransmitters from the extracellular space [120,121]. Any disruption in glutamate homeostasis can lead to detrimental impacts on the brain. It is important to note that a classical feature of thiamine deficiency is the astrocytic loss of these transporters [122]. During thiamine deficiency, astrocytes downregulate their expression of EAAT1/EAAT2 which leads to a dramatic increase in glutamate levels within the extracellular space of the brain. The disruption causes a neuron-excitatory toxic environment and ultimately brain death [120-122]. To support this, an animal study demonstrated a downregulation of the glutamate transporter proteins in the cerebral cortex of WK [120-122]. Oxidative stress mediated by nitric oxide synthase and 4-hydroxynonenal (HNE) is another common abnormality seen in thiamine deficiency [122,123]. It causes dysfunctions in the glutamate transporters and astrocytic integrity which further contribute to the loss of cerebral cortex brain tissue [122,123]. Glial cells contribute to CNS tissue loss seen in WK in a multifactorial manner. It is important to note that although TDP-43 was not observed or studied in these studies there is potential as a few studies showed amyloid plaque accumulation in mice [124]. Therefore, further investigation should be done to detect associations between WK and TDP-43 involvement in amyloid plaque.

Von Hippel-Lindau (VHL) Protein

VHL protein, a tumor suppressor, is part of the cullin-2 (CUL2) ring complex that functions in the ubiquitin ligase of fragmented forms of TDP-43 [125]. VHL substrate binding protein serves two roles; it promotes the degradation of misfolded TDP-43 with CUL2 and when overexpressed, stabilizes aggregates of TDP-43 at the juxtanuclear protein quality control center (JUNQ) [125]. VHL/CUL2 E3 complex can recognize and bind to misfolded TDP-43 in the CNS, Purkinje calls, Golgi type II cells as well as the dentate nucleus of the cerebellum. The complex is also seen in the maturation of oligodendrocytes [125]. An imbalance in VHL/CUL2, or abnormal interaction between VHL and misfolded TDP-43, as seen in overexpression of VHL, led to cytoplasmic inclusions in oligodendrocytes [125]. As previously mentioned, since VHL has a role in the normal maturation of oligodendrocytes, an imbalance in such a complex would interfere with this process, causing oligodendrocyte dysfunction due to cytosolic inclusions and in turn neurodegeneration [125]. TDP-43 levels remain unchecked in this disease, however, further investigations could be necessary to confirm if cytosolic inclusions of such oligodendrocytes are composed of just aggregates of TDP-43 alone or other proteinopathies causing cellular toxicity and dysfunction.

*Barth Syndrome (BS)* 

BS is an X-linked recessive disorder that has mutations in the tafazzin (TAZ) gene, which is responsible for cardiolipin remodeling from monolysocardiolipin, which has major effects on skeletal muscle and cardiac muscles [126]. Cardiolipin is a mitochondrial exclusive phospholipid important in maintaining mitochondrial structure, membrane fluidity, cellular signaling, and cellular metabolism [126]. A mutation to TAZ causes an imbalance in the cardiolipin and monolysocardiolipin ratio and ultimately affects normal mitochondrial functions [126]. This is a new area of focus for neurodegenerative diseases with abnormal lipid profiles including AD, ALS, PD, and FTLD [126]. They observed that TAZkd mice have reduced protein expression along with decreased cardiolipin and a substantial increase in monolysocardiolipin compared to controls [126]. In turn, reactive oxygen species production was noted to be increased, which is detrimental to cellular viability, especially to oligodendrocytes that are sensitive to oxidative stress. This leads to the activation of the neuroinflammatory response starting with microglia which was observed in TAZkd mice [126]. One of the roles of cardiolipin studied in a neuronal cell is that it translocates from the inner mitochondrial membrane to the outer mitochondrial membrane in response to mitochondrial damage [126]. This functions in mitophagy to activate autophagosome and lysosome fusion to eliminate the damaged mitochondria [126]. Studies show that over-expression of TDP-43 activates glycogen synthase kinase-3β (GSK-3β) which is an important regulator of mitochondrial homeostasis including Ca^2+^ and phospholipid exchange [126]. The interaction between autophagosome and lysosome activation could be an important signaling mediator in the induction of microglia and gliosis [126]. With a new focus on cardiolipin in neuronal cells and neurodegenerative disease, further research could show its association with TDP-43 activation of ER-mitochondria metabolism, translocation of TDP-43 to mitochondria, and its effect on glial cells in TDP-43 proteinopathies.

*Pompe’s Disease (PD)* 

PD is a type of glycogen storage disease caused by the deficiency of 𝛼-glucosidase (GAA), which leads to a toxic accumulation of glycogen in the brain and heart [127]. The accumulation is caused by both an abnormal lysosome and phagosome resulting in dysfunctional autophagy [127]. Primary manifestations of PD include weakness and cardiomyopathy [127]. Focusing on the nervous system, in particular, glycogen accumulation has been seen in the glial cells located in the brain cortex and brainstem, as well as anterior horn cells of the spinal cord [127]. A study was performed by Lee et al. (2006) where they examined 20 infantile cases of PD at autopsy and concluded that signs of brain involvement in infantile-onset are unclear [127]. They found two subjects' magnetic resonance imaging (MRI) findings conclusive for the widening of the anterior horns of the lateral ventricles and central cortical atrophy [127]. The hypothesis for this uncertainty is believed to be that patients with PD die before brain illness can be investigated [127]. Increased glycogen levels in PD are found to be predominantly in glial cells including small blood vessels in the striatum and neocortex. Normally, low levels of glycogen stores in astrocytes produce lactate as a source of energy that is transported to neurons that are active via monocarboxylate transporter MCT2 [127]. Glycogen in astrocytes synthesizes glutamine, therefore an imbalance in glycogen in turn causes an imbalance in glutamine to glutamate neurotransmission [128]. The accumulation of glycogen in astrocytes seen in PD causes loss of normal function [128]. Astrocyte neurotransmission function is altered causing problems in the CNS, specifical abnormalities in learning and memory as well as seizures [128]. The role of TDP-43 has yet to be studied in relation to PD specifically, however, it has been studied in several other neurodegenerative diseases [128]. A possible relationship between TDP-43 and PD might be present as glycogen accumulation interferes with normal autophagy. More research should be done to investigate whether accumulated glycogen alone plays a role in the absence of phagolysosome fusion or if increased stores of glycogen accumulation lead to accumulation of TDP-43, where its fragments can not be degraded since those with PD already have dysfunctional phagolysosome trafficking. If such processing exists, where TDP-43 is also accumulated, this can be another cause of glial cell damage seen in this disease.

*Sturge Weber (SW)*****

SW is a rare neurocutaneous disorder caused by a somatic mutation in the guanine nucleotide-binding protein G (GNAQ) gene [129]. Individuals with SW manifest with abnormalities in the brain, skin, and/or eyes. The most common pathological finding is leptomeningeal angiomatosis, however recent research has shown that cerebral cortical and white matter abnormalities play a role in disease presentation [129]. Sundaram et al. (2017) hypothesized that SW can present with or without detectable leptomeningeal angiomatosis [129]. They investigated the brain specimens of nine children with SW according to leptomeningeal angiomatosis and non-leptomeningeal angiomatosis groups. They found that all nine with leptomeningeal angiomatosis and seven out of nine with non-leptomeningeal angiomatosis had a mutation in the GNAQ gene [130,131]. This mutation does not only cause vascular abnormalities but can also affect brain parenchyma as well as hypoplastic blood vessels, which showed cerebral gliosis and calcifications within the gray and white matter [130,131]. The calcifications are believed to be a possible cause of anoxic injury to endothelial, perithelial as well as injury to the mitochondria of glial cells [130,131]. Anoxic injury to these vital cells compromises their function, leading to neuronal apoptosis and astrogliosis. The calcification can increase blood vessel permeability, and then atrophy [130,131], secondary to reactive changes in glial cell response particularly microglia to this disease’s pathogenesis.

Microglia are extremely sensitive to their microenvironment and as innate immune cells of the CNS, and under normal conditions, they express complement receptor type 3 (CR3) as well as the major histocompatibility complex (MHC I) that are involved in the immune response [131,132]. Any abnormalities, such as anoxia, cause upregulation of CR3 and MHC I. Further, microglial exhibits MHC II that is not seen in normal conditions [131,132]. Upregulation of these factors is warranted as phagocytosis is the end goal of the immune response by microglia in the CNS [131,132]. Calcifications and induced anoxic injury to glial cells lead to stasis and dystrophic changes causing abnormal cerebral vessel permeability [131,132]. Also, during normal conditions, hypoxia-inducible factor (HIF) can be ubiquitinated and degraded in proteasomes, however, in a hypoxic state, HIF is not degraded and therefore rapidly accumulates [131,132]. As a result of the accumulation of HIF, intrinsic regulators stimulate receptor and ligand expression, inducing vascular endothelial growth factor (VEGF-1 and 2) [133]. Per Comati et al., patients with SW had an increased number of VEGF mRNA expressions in neurons and glial cells [133]. Even though the specific role of TDP-43 in the pathogenesis of SW specifically has not been studied, we suggest further studies be done to investigate a possible correlation [133]. Abnormal neuronal and glial cell interactions have been studied. So far there is evidence that accumulation of TDP-43 in the cytosol may activate glial cells, therefore further investigation is highly recommended to reveal whether glial cells are activated due to neuronal pathology or due to accumulations of TDP-43 in the neurons [133].

## Conclusions

TDP-43 and glial cells are involved in various pathophysiology with different mechanisms elaborated above. The signaling cascades among both components contribute to multiple neurological disorders. Therefore, further investigations could expand on TDP-43’s function as a second messenger and its role that is involved in glial cell activation and maintenance of the neuroinflammatory cascade. This results in the rapid progression of TDP-43 proteinopathies. The toxic aggregation of TDP-43 in the cytosol has been associated with a majority of the proteinopathies that have been studied. Accumulation of TDP-43 has been observed in microglia, astrocytes, and oligodendrocytes/oligodendrocyte progenitor cells in these diseases. The elucidation of the mechanism of TDP-43 and glial cell activation behind the pathogenesis of neurodegenerative diseases is the first step in unveiling the accurate pathophysiology of different phenotypes of TDP-43 proteinopathies. In particular, this reveals the pleiotropy that is seen within each disease.
